# Mesenchymal Stem Cells Combined with a P(VDF-TrFE)/BaTiO_3_ Scaffold and Photobiomodulation Therapy Enhance Bone Repair in Rat Calvarial Defects

**DOI:** 10.3390/jfb14060306

**Published:** 2023-06-01

**Authors:** Leticia Faustino Adolpho, Larissa Mayra Silva Ribeiro, Gileade Pereira Freitas, Helena Bacha Lopes, Maria Paula Oliveira Gomes, Emanuela Prado Ferraz, Rossano Gimenes, Marcio Mateus Beloti, Adalberto Luiz Rosa

**Affiliations:** 1 Bone Research Lab, School of Dentistry of Ribeirão Preto, University of São Paulo, Ribeirão Preto 14040-904, SP, Brazil; leticia.adolpho@usp.br (L.F.A.); gileade@ufg.br (G.P.F.); helena.lopes@usp.br (H.B.L.); maria.paula.gomes@usp.br (M.P.O.G.); emanuelaferraz@usp.br (E.P.F.); mmbeloti@usp.br (M.M.B.); 2 Institute of Physics and Chemistry, University of Itajubá, Itajubá 37500-903, MG, Brazil; larissamayra@unifei.edu.br (L.M.S.R.); rossano@unifei.edu.br (R.G.); 3 School of Dentistry, Federal University of Goiás, Goiânia 74605-020, GO, Brazil

**Keywords:** bone repair, electrospinning, laser therapy, mesenchymal stem cell, PVDF scaffold

## Abstract

Background: Tissue engineering and cell therapy have been the focus of investigations on how to treat challenging bone defects. This study aimed to produce and characterize a P(VDF-TrFE)/BaTiO_3_ scaffold and evaluate the effect of mesenchymal stem cells (MSCs) combined with this scaffold and photobiomodulation (PBM) on bone repair. Methods and results: P(VDF-TrFE)/BaTiO_3_ was synthesized using an electrospinning technique and presented physical and chemical properties suitable for bone tissue engineering. This scaffold was implanted in rat calvarial defects (unilateral, 5 mm in diameter) and, 2 weeks post-implantation, MSCs were locally injected into these defects (*n* = 12/group). Photobiomodulation was then applied immediately, and again 48 and 96 h post-injection. The μCT and histological analyses showed an increment in bone formation, which exhibited a positive correlation with the treatments combined with the scaffold, with MSCs and PBM inducing more bone repair, followed by the scaffold combined with PBM, the scaffold combined with MSCs, and finally the scaffold alone (ANOVA, *p* ≤ 0.05). Conclusions: The P(VDF-TrFE)/BaTiO_3_ scaffold acted synergistically with MSCs and PBM to induce bone repair in rat calvarial defects. These findings emphasize the need to combine a range of techniques to regenerate large bone defects and provide avenues for further investigations on innovative tissue engineering approaches.

## 1. Introduction

Bone is a specialized connective tissue that exhibits great capacity to repair and regenerate when damaged, which may be surpassed by the extension of the lesion, demanding further interventions to achieve restoration in terms of content, anatomy and function [1,2,3]. The concepts of tissue engineering and cell therapy have been extensively employed to investigate and develop new approaches to treat challenging bone defects [4,5,6,7,8]. In this scenario, the combination of biomaterials and cells offers a promising alternative to autogenous bone graft, the current gold standard material [9,10].

Among a plethora of biomaterials, piezoelectric materials are of interest as they can transduce electrical stimuli to physiological systems in response to events such as cell migration and due to the susceptibility of bone cells to this property [11,12,13]. The piezoelectric composite poly(vinylidene-trifluoroethylene)/barium titanate (P(VDF-TrFE)/BaTiO_3_) favors osteoblast differentiation of human alveolar bone-derived cells compared with polytetrafluoroethylene (PTFE) [14,15]. Additionally, the membrane of P(VDF-TrFE)/BaTiO_3_ induces more bone formation than PTFE in calvarial defects of healthy and osteoporotic rats [16,17]. Such beneficial effects on bone repair are due, at least in part, to the bone resorption inhibition triggered by P(VDF-TrFE)/BaTiO_3_ through the regulation of a microRNA-34a/receptor activator of nuclear factor kappa B ligand (RANKL) circuit [18].

The good bone response to P(VDF-TrFE)/BaTiO_3_ composite made it a candidate to be combined with cells to enhance bone repair. Mesenchymal stem cells (MSCs) combined with a P(VDF-TrFE)/BaTiO_3_ membrane produce more bone repair than the membrane alone when implanted in rat calvarial defects under healthy and osteoporotic conditions [19,20]. The increment in bone repair observed when cells and P(VDF-TrFE)/BaTiO_3_ were combined suggests that fine-tuning the composite modification and cell response stimulation could result in complete regeneration of the calvarial defect.

Considering bone tissue engineering, scaffolds are more suitable than membranes, and an ideal scaffold should exhibit properties and an architecture that mimic the extracellular matrix, creating a favorable environment for tissue growth [21,22]. Scaffolds can be created using an electrospinning technique, which is a simple and low-cost method of producing fibers at the micro and nanoscale that generate structures with increased surface area based on the material volume [23,24]. Thus, this technique may be employed to produce P(VDF-TrFE)/BaTiO_3_ scaffolds that may favor bone formation. Regarding cell response stimulation, the use of inflammatory mediators, drugs and specific culture conditions may increase the efficiency of MSCs in regenerative medicine procedures [25,26]. In this context, photobiomodulation (PBM) therapy acts on several signaling pathways that regulate cellular events such as proliferation and differentiation, which are involved in bone formation [27,28]. PBM therapy restores the osteogenic capacity of MSCs derived from diabetic rats and enhances bone formation in rat dental alveolus filled with hydroxyapatite [29,30]. Additionally, PBM increases bone repair in critical size defects treated with either MSCs derived from dental pulp combined with hydrogel or MSCs from adipose tissue combined with decellularized bone matrix [31,32].

Although MSCs can be combined with a P(VDF-TrFE)/BaTiO_3_ scaffold as well as PBM therapy to enhance bone formation, the combination of these three approaches, cells, scaffolds and PBM, is underexplored in the field of regenerative medicine. Thus, this study aimed to synthesize and characterize a P(VDF-TrFE)/BaTiO_3_ scaffold produced using an electrospinning technique and evaluate the effect of MSCs combined with this scaffold and submitted to PBM therapy on bone repair.

## 2. Materials and Methods

### 2.1. Synthesis of the P(VDF-TrFE)/BaTiO_3_ Scaffold

The P(VDF-TrFE)/BaTiO_3_ (90/10, % in volume) composite was obtained by dissolving the copolymer P(VDF-TrFE) (Arkema Piezotech, Pierre-Benite Cedex, France) dispersed in N, N-dimethylformamide (DMF, Sigma-Aldrich, Saint Louis, MO, USA) and acetone (Synth, Diadema, SP, Brazil) at a ratio of 7:3 at 50 °C in a water bath for 3 h. After the complete dissolution of the copolymer, BaTiO_3_ powder (Sigma-Aldrich) was added to the solution. The copolymer/solvent ratio was 20 g/100 mL. The resulting solution was homogenized using an ultrasonic processor VCX 750 (Sonics & Materials Inc., Newtown, CT, USA) for 6 min in a water–ice bath. The solution was electrospun using a setup composed of a NE-300 syringe pump (New Era, Farmingdale, NY, USA), adjusted to a flow rate of 1500 µL/h, 100 mm from the collector. The voltage applied to the needle (inner diameter of 1.20 mm) was 12 kV using a high-power supply ES40P-5W (Gamma High Voltage, Ormond Beach, FL, USA). The temperature was kept at 25 °C with 42% humidity during electrospinning and the deposition time required to obtain scaffold plates with 0.6 mm in thickness was 30 min. The fibers were collected on nonstick paper and maintained in a vacuum oven for 24 h at 30 °C to ensure that any residual solvent evaporated. Discs of P(VDF-TrFE)/BaTiO_3_ scaffold were cut with a hole punch (5 mm in diameter) in liquid nitrogen bath to preserve the three-dimensional structure on the edges. Prior to implantation, scaffolds were submitted to ethylene oxide sterilization.

### 2.2. P(VDF-TrFE)/BaTiO_3_ Scaffold Characterization

The fiber morphology was examined using scanning electron microscopy (SEM, Phenom ProX, Thermo Fisher Scientific, Waltham, MA, USA). The fiber diameter was estimated in the SEM micrographs using ImageJ software (National Institutes of Health, Bethesda, MD, USA). The pore size distribution was evaluated via microtomographic (µCT) analysis using the SkyScan 1172 system (Bruker-Skyscan, Kontich, Belgium) and the three-dimensional reconstructions were generated using the NRecon Cluster software (Micro Photonics Inc., Allentown, PA, USA). The mean centric linear roughness (*R_a_*) was measured in 5 locations of 3 samples employing three-dimensional images generated by the Phenom ProX SEM software. The elemental analysis was performed on two different areas of the scaffold (fibers and particles) using energy-dispersive X-ray spectroscopy (SEM-EDS, Superscan SSX-550, Shimadzu Corp., Kyoto, Japan) at an acceleration voltage of 20 kV, a working distance of 8 mm and an integration time of 50 s. The P(VDF-TrFE)/BaTiO_3_ wettability was assessed using a sessile Easy Drop Shape Analyzer (Krüss Scientific, Hamburg, Germany) through contact angle measurements and compared with P(VDF-TrFE) and PVDF. The contact angle was measured at 5 positions of 5 samples (*n* = 5) using 10 µL of deionized water drop, immediately after drop placement, at 18 °C. Additionally, the contact angle on P(VDF-TrFE)/BaTiO_3_ was measured at 5 positions of 5 samples (*n* = 5) every 10 min up to 40 min.

### 2.3. Evaluation of Bone Repair

#### 2.3.1. Animals

This study involved 60 male Sprague Dawley rats weighing 150–200 g according to the rules of the Committee of Ethics in Animal Research of the School of Dentistry of Ribeirão Preto (Protocol # 0031/2021; date of approval: 11/10/2021).

#### 2.3.2. Isolation and Culture of MSCs

MSCs were harvested from bone marrow of the femurs of 12 rats and cultured in non-inducing culture medium until they reached 70% confluence, as previously described [20]. First-passage MSCs were enzymatically detached and directly injected into rat calvarial defects, as described below. The culture medium was changed every 48 h.

#### 2.3.3. Creation and Treatment of Calvarial Defects

Forty-eight rats were anesthetized with ketamine (75 mg/kg, intraperitoneal; Agener União, Embu-Guaçu, SP, Brazil) and xylazine (6 mg/kg, intraperitoneal; Calier, Juatuba, MG, Brazil) and a unilateral 5-mm-diameter defect was created using a trephine drill (Neodent, Curitiba, PR, Brazil). Then, the defects were implanted with the scaffold and the skin was sutured with mononylon 4.0 (Ethicon Ltd.a, São José dos Campos, SP, Brazil). Two weeks post-calvarial defect creation and scaffold implantation, the animals were randomly grouped (*n* = 12 per group) according to the treatment of bone defects: (1) only the scaffold (Scaffold); (2) the scaffold combined with MSCs (Scaffold + MSCs); (3) the scaffold combined with PBM therapy (Scaffold + PBM); and (4) the scaffold combined with MSCs and PBM therapy (Scaffold + MSCs + PBM). The rats were anesthetized and 5 × 10^6^ MSCs in 50 µL of phosphate-buffered saline (PBS, Gibco-Life Technologies) were locally injected into each defect, except for those treated with the scaffold without cells, which were injected with 50 µL of PBS (Gibco-Life Technologies). The PBM was based on the local applications of gallium–aluminum–arsenide laser (GaAIAs, Photon III, DMC, São Carlos, SP, Brazil) in continuous contact and punctual operation mode in four points of the calvarial defect, one central and three equidistant around the defect. Irradiations were performed immediately after, as well as 48 and 96 h post-injection, according to the following parameters: wavelength: 808 nm, power: 40 mW, power density: 1.42 W/cm^2^, energy density: 4 J/cm^2^, irradiation time: 3 s, energy per point: 0.12 J, and spot area: 0.028 cm^2^ [33,34]. Four weeks post injection, the animals were euthanized. The calvarias were harvested and fixed in 10% buffered formalin to evaluate the newly formed bone.

#### 2.3.4. µCT Analysis

The µCT analysis was carried out by a single blinded operator using the SkyScan 1172 system (Bruker-Skyscan) and the three-dimensional reconstructions were created using NRecon Cluster software (Micro Photonics Inc.) as previously described [19]. Bone volume (BV, mm^3^), percentage of bone volume (BV/TV, %), bone surface (BS, mm^2^), trabecular number (Tb.N, 1/mm), trabecular separation (Tb.Sp, mm) and bone mineral density (BMD, g/cm^3^) were evaluated in the region of interest: the 5 mm diameter of the calvarial defect [35].

#### 2.3.5. Histological Analysis

After µCT analysis, undecalcified calvariae were dehydrated, embedded in resin (LR White Hard Grade, London, UK) and sectioned using the Exakt cutting system (Exakt, Norderstedt, Germany) to produce two sections per sample. The 150-μm-thick sections were mounted on glass slides and polished to a thickness of 70 μm. The sections were stained with Stevenel’s blue (Sigma-Aldrich) for 15 min at 55 °C and alizarin red (Sigma-Aldrich) for 2 min at room temperature or toluidine blue for 20 min at room temperature (Merck, Darmstadt, Germany). The images were obtained using a light microscope (Axioskop 40, Carl Zeiss Inc., Oberkochen, Germany) coupled with a digital camera (Axiocam ICc3, Carl Zeiss).

### 2.4. Statistical Analysis

The wettability data from P(VDF-TrFE)/BaTiO_3_, P(VDF-TrFE) and PVDF (*n* = 5) were compared by one-way ANOVA followed by Duncan’s new multiple range test and from P(VDF-TrFE)/BaTiO_3_ over time (*n* = 5) by repeated measures ANOVA followed by Tukey’s post hoc test. The data from morphometric parameters (*n* =12) were compared by one-way ANOVA followed by Duncan’s new multiple range test and by Pearson’s correlation coefficient. The data were expressed as mean ± standard deviation (*p* ≤ 0.05).

## 3. Results

### 3.1. P(VDF-TrFE)/BaTiO_3_ Scaffold Characterization

**Figure 1:**
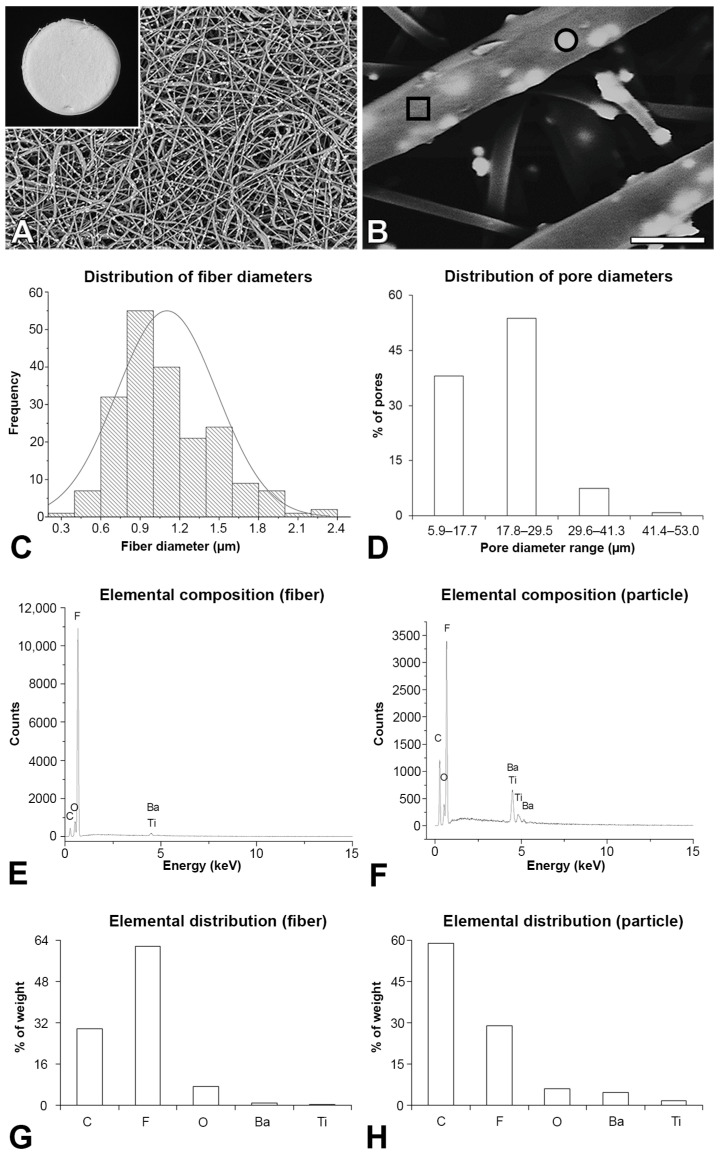
P(VDF-TrFE)/BaTiO_3_ scaffold characterization. Macroscopic view ((**A**), inset) and scanning electron microscopy of the P(VDF-TrFE)/BaTiO_3_ scaffold showing the fiber distribution (**A**) and details of the fibers with particles (**B**). Distribution of the fiber diameter (**C**) and pore size (**D**) of the P(VDF-TrFE)/BaTiO_3_ scaffold. Energy-dispersive X-ray spectroscopy of the P(VDF-TrFE)/BaTiO_3_ scaffold showing elemental composition of the fiber ((**E**), square in (**B**)) and the particle ((**F**), circle in (**B**)), and elemental distribution of the fiber (**G**) and the particle (**H**). Scale bar: (**A**) = 80 μm; (**A**) (inset) = 3.75 mm; (**B**) = 2 μm.

The electrospun P(VDF-TrFE)/BaTiO_3_ fibers were uniform and continuous, without interruptions or beads along them (Figure 1A). It was possible to see particles as aggregates uniformly distributed on the fibers (Figure 1B). The average fiber diameter was 1.10 ± 0.38 µm (Figure 1C). The pore sizes were distributed in four ranges, with approximately 50% of the pores ranging from 17.68 to 29.47 µm (Figure 1D), and the average *Ra* was 0.869 ± 0.03 µm. The P(VDF-TrFE)/BaTiO_3_ scaffold was composed of C, F, O, Ba and Ti regardless of the analyzed area, the fiber (Figure 1E) or the particle (Figure 1F). The elemental distribution varied according to the area analyzed with a higher percentage of F in the fiber (Figure 1G) and of C, Ba and Ti in the particle (Figure 1H).

**Figure 2:**
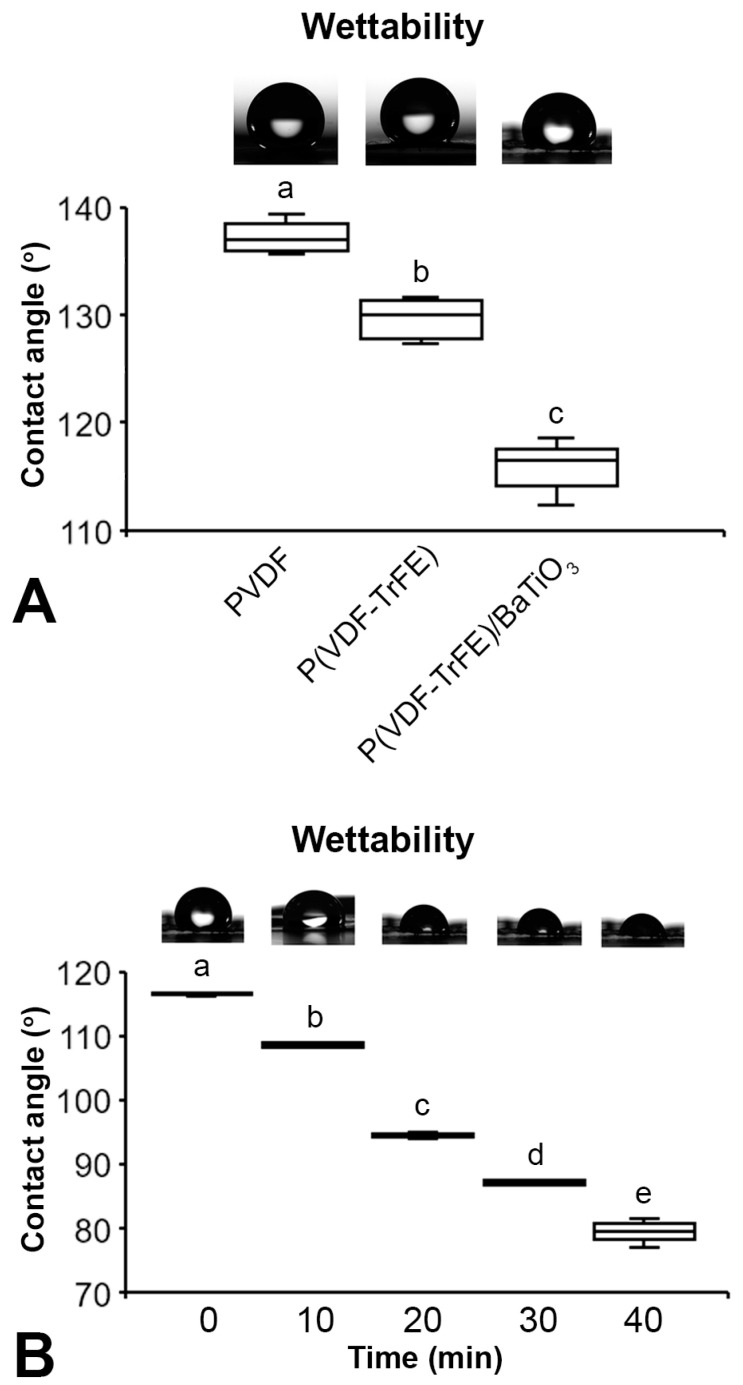
P(VDF-TrFE)/BaTiO_3_ scaffold characterization. Wettability of the P(VDF-TrFE)/BaTiO_3_ compared with P(VDF-TrFE) and PVDF measured via the contact angle immediately after placing the deionized water drop (**A**) and time course of the contact angle measured on P(VDF-TrFE)/BaTiO_3_ (**B**). The data are presented as mean ± standard deviation (*n* = 5). Different letters represent statistically significant differences among P(VDF-TrFE)/BaTiO_3_, P(VDF-TrFE) and PVDF ((**A**), a–c, *p* < 0.001) and time ((**B**), a–e, *p* < 0.002).

The contact angle was lower on P(VDF-TrFE)/BaTiO_3_ compared with either P(VDF-TrFE) or PVDF, and lower on P(VDF-TrFE) than PVDF (*p* < 0.001 for all comparisons) (Figure 2A). The contact angle progressively decreased (*p* < 0.002 for all comparisons) on P(VDF-TrFE)/BaTiO_3_ over time, but it is still possible to observe the drop even after 40 min (Figure 2B).

### 3.2. Evaluation of Bone Repair

**Figure 3:**
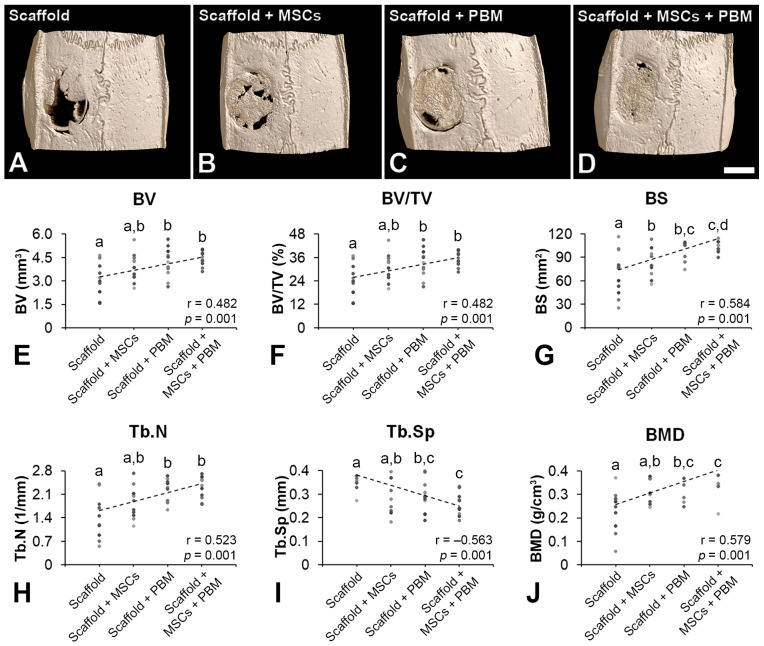
Evaluation of bone repair. Three-dimensional microtomographic reconstructions (**A**–**D**) of bone formation in rat calvarial defects treated with P(VDF-TrFE)/BaTiO_3_ scaffold (Scaffold, (**A**)); scaffold combined with bone marrow-derived mesenchymal stem cells (Scaffold + MSCs, (**B**)); scaffold combined with photobiomodulation (Scaffold + PBM, (**C**)); and scaffold combined with MSCs and PBM (Scaffold + MSCs + PBM, (**D**)). Morphometric parameters bone volume (BV, (**E**)), percentage of bone volume (BV/TV, (**F**)), bone surface (BS, (**G**)), trabecular number (Tb.N, (**H**)), trabecular separation (Tb.Sp, (**I**)) and bone mineral density (BMD, (**J**)) evaluated in the region of interest, the 5 mm diameter of the calvarial defect. The data are presented as mean ± standard deviation (*n* = 12). Different letters (a–d) represent statistically significant differences among the treatments ((**E**–**J**), *p* ≤ 0.05). Scale bar: (**A**–**D**) = 2.50 mm.

The three-dimensional reconstructions indicated more bone repair in defects treated with Scaffold + MSCs + PBM followed by Scaffold + PBM, Scaffold + MSCs and Scaffold (Figure 3A–D), and the morphometric parameters confirmed this finding (Figure 3E–J). The BV was higher in defects treated with Scaffold + MSCs + PBM and Scaffold + PBM compared with Scaffold (*p* ≤ 0.05) and showed a positive correlation with treatments (r = 0.482, *p* = 0.001) (Figure 3E). The BV/TV was higher in defects treated with Scaffold + MSCs + PBM and Scaffold + PBM compared with Scaffold (*p* ≤ 0.05) and presented a positive correlation with treatments (r = 0.482, *p* = 0.001) (Figure 3F). The BS was higher in defects treated with Scaffold + MSCs + PBM compared with Scaffold + MSCs and Scaffold (*p* ≤ 0.05). The BS was higher in defects treated with Scaffold + PBM and Scaffold + MSCs compared with Scaffold (*p* ≤ 0.05) and exhibited a positive correlation with treatments (r = 0.584, *p* = 0.001) (Figure 3G). The Tb.N was higher in defects treated with Scaffold + MSCs + PBM and Scaffold + PBM compared with Scaffold (*p* ≤ 0.05) and showed a positive correlation with treatments (r = 0.523, *p* = 0.001) (Figure 3H). The Tb.Sp was lower in defects treated with Scaffold + MSCs + PBM compared with Scaffold + MSCs and Scaffold (*p* ≤ 0.05). The Tb.Sp was lower in defects treated with Scaffold + PBM compared to Scaffold (*p* ≤ 0.05) and presented a negative correlation with treatments (r = −0.563, *p* = 0.001) (Figure 3I). The BMD was higher in defects treated with Scaffold + MSCs + PBM compared with Scaffold + MSCs and Scaffold (*p* ≤ 0.05). The BMD was higher in defects treated with Scaffold + PBM compared with Scaffold (*p* ≤ 0.05) and exhibited a positive correlation with treatments (r = 0.579, *p* = 0.001) (Figure 3J).

**Figure 4:**
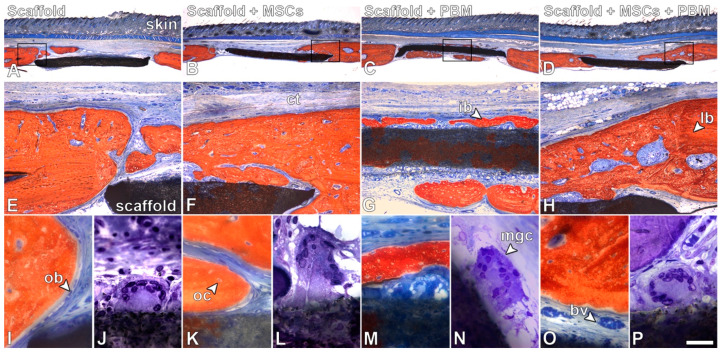
Evaluation of bone repair. Light microscopy of bone formation in rat calvarial defects treated with P(VDF-TrFE)/BaTiO_3_ scaffold (Scaffold, (**A**,**E**,**I**)), Scaffold + MSCs (**B**,**F**,**K**), Scaffold + PBM (**C**,**G**,**M**) and Scaffold + MSCs + PBM (**D**,**H**,**O**). Multinucleated giant cells were observed in close contact with the scaffold surface in all defects, irrespective of treatment (**J**,**L**,**N**,**P**). Stevenel’s blue and alizarin red (**A**–**I**,**K**,**M**,**O**) and toluidine blue (**J**,**L**,**N**,**P**) staining. Squares in (**A–D**) are represented in (**E–H**). Scale bar: (**A**–**D**) = 1.25 mm; (**E**–**H**) = 200 μm; (**I**,**K**,**M**,**O**) = 50 μm; (**J**,**L**,**N**,**P**) = 20 μm. bv = blood vessel; ct = connective tissue; ib = immature bone; lb = lamellar bone; mgc = multinucleated giant cell; ob = osteoblast; oc = osteocyte.

The histological sections stained with Stevenel’s blue and alizarin red showed the presence of bone in the edges of the defects and in close contact with the scaffold, irrespective of the treatments (Figure 4A–I,K,M,O). The newly formed bone exhibited characteristics of healthy tissue with areas of immature and lamellar bone, and the presence of osteoblasts, osteocytes and blood vessels, without signs of adverse reactions. Multinucleated giant cells were observed in close contact with the scaffold in histological sections stained with toluidine blue in all of the evaluated groups (Figure 4J,L,N,P).

## 4. Discussion

Treating large bone defects still represents a significant clinical challenge in orthopedics and oral and maxillofacial surgery, and several strategies focused on tissue engineering and cell therapy have been proposed to manage this issue. As scaffolds, cells and PBM therapy effectively favor bone repair, a synergistic effect is expected when combining them during bone formation. Here, we synthesized a P(VDF-TrFE)/BaTiO_3_ scaffold using an electrospinning technique with physical and chemical properties that make it suitable to be employed in bone tissue engineering. Then, we demonstrated that the combination of this scaffold with MSCs and PBM therapy is a good strategy to enhance bone formation in a rat calvarial defect model.

Considering that biomaterials should promote a favorable environment for cell adhesion and growth, the properties of scaffolds produced using an electrospinning technique should affect these cell events, with a positive effect on bone formation. As expected, the P(VDF-TrFE)/BaTiO_3_ scaffold was composed of C, F, O, Ba and Ti regardless of the analyzed area; however, the elemental distribution varied with the fibers exhibiting a higher percentage of F and the particles more C, Ba and Ti. The fiber diameters of the P(VDF-TrFE)/BaTiO_3_ scaffold synthesized here are in the same range as a biodegradable polyhydroxybutyrate composite that allowed MSC adhesion and spreading [36]. Regarding the pore sizes, the majority ranged from 17 to 29 μm; this did not allow cell infiltration, since MSCs vary from 18 to 30 μm [37]. Such characteristics could explain the bone formation observed only on the P(VDF-TrFE)/BaTiO_3_ scaffold surface, making the increasing pore size relevant to further investigations. The surface wettability is one of the most important factors determining cell adhesion. The contact angle on PVDF and P(VDF-TrFE)-based materials is related to the hydrophobicity of fluorinated polymers, as alkyl and fluorinated alkyl groups exhibit low interaction energy with water [38]. The contact angle on PVDF was higher than on P(VDF-TrFE) due to the presence of free alkyl groups in the PVDF structure. The addition of BaTiO_3_ reduced the contact angle thanks to its super-hydrophilic characteristic [39]. The detected contact angle of 79° after 40 min suggests that the P(VDF-TrFE)/BaTiO_3_ scaffold may favor cell adhesion, since effective adhesion occurs on surfaces with water contact angles ranging from 40 to 80° [40]. Because the presence of nanoparticles affects the thermal and mechanical properties of biomaterials, the addition of BaTiO_3_ may have modified these scaffold features, potentially influencing the bone response [41]. Thus, further characterizations of the P(VDF-TrFE)/BaTiO_3_ scaffold should consider the use of molecular dynamics simulation, a low-risk/-cost approach compared with experimental methods, to evaluate its thermal and mechanical properties [41,42]. Together, the features of the P(VDF-TrFE)/BaTiO_3_ scaffold seem adequate for a biomaterial to be employed in bone tissue engineering approaches.

Several studies have demonstrated the osteogenic potential of P(VDF-TrFE) composites in different in vitro and in vivo models. P(VDF-TrFE)/BaTiO_3_ membrane favors osteoblast differentiation compared with PTFE, and P(VDF-TrFE)/boron nitride nanotubes promote differentiation of SaOS-2 osteoblast-like cells [14,15,43]. Additionally, P(VDF-TrFE)/BaTiO_3_ membranes induce more bone formation than PTFE when implanted in calvarial defects of either healthy or osteoporotic rats [16,17,44]. In agreement with these findings, we showed that calvarial defects treated with the P(VDF-TrFE)/BaTiO_3_ scaffold and PBS injection exhibit significant bone repair compared with defects without the scaffold that were injected with PBS, which are generally filled with connective tissue as previously demonstrated [45,46]. In addition to upregulating osteoblast differentiation, P(VDF-TrFE)/BaTiO_3_ enhances bone formation by inhibiting bone resorption through the regulation of microRNA-34a/RANKL crosstalk [18].

Although good results were observed in defects treated with the P(VDF-TrFE)/BaTiO_3_ scaffold, the combination of scaffolds and cells proved to be better in terms of promoting bone repair. Indeed, polymer/hydroxyapatite scaffolds combined with MSCs were more effective than the scaffold alone in the repair of rat calvarial defects [47,48]. Additionally, P(VDF-TrFE)/BaTiO_3_ membrane combined with osteoblasts differentiated from bone marrow MSCs promotes more bone formation than the membrane alone in calvarial defects of healthy animals; this was also true of MSCs in osteoporotic rats [19,20]. Corroborating these findings, we demonstrated that MSCs combined with the P(VDF-TrFE)/BaTiO_3_ scaffold resulted in more bone formation compared with the scaffold alone. As the cells stay in the bone defect for approximately 25 days in an experimental model such as this one, the increased bone repair induced by the presence of MSCs could be related to their ability to home and integrate into damaged tissues and provide osteogenic and immunomodulatory effects [19,49,50].

To increase the bone repair induced by the P(VDF-TrFE)/BaTiO_3_ scaffold combined with MSCs, the PBM was employed as an adjunctive therapy, since its stimulatory effects on host stem cell recruitment, osteoblast differentiation and bone formation have been demonstrated in cell culture and animal models [51,52,53]. The combination of a collagen membrane with PBM therapy using the same irradiation parameters employed here induced more bone formation than the membrane alone in calvarial defects of osteoporotic rats [34]. In keeping with this, our results showed that PBM therapy increases bone repair in defects implanted with the P(VDF-TrFE)/BaTiO_3_ scaffold compared with non-irradiated defects. Notably, PBM promoted slightly more bone formation than MSCs when combined with the scaffold, although a non-statistically significant difference was detected. In contrast, PBM did not affect bone repair in calvarial defects of osteoporotic rats implanted with P(VDF-TrFE)/BaTiO_3_ membranes, which could be partially explained by the differences in irradiation parameters. This supports the relevance of stablishing a precise protocol for PBM therapy application [54].

Different combinations of two out of these three approaches have been the subject of several studies; however, the combination of all three—biomaterials, cells and PBM therapy—is underexplored in the field of bone regeneration. The PBM application in calvarial defects treated with adipose-derived MSCs encapsulated in methacrylated gelatin hydrogels increased bone formation; this also occurred in defects treated with MSCs from dental pulp encapsulated in an injectable BMP-4-loaded hydrogel [32,55]. Here, we observed a progressive increment in bone formation which was confirmed by the correlation between treatments for all evaluated morphometric parameters. This increase was greater than that for defects treated with the P(VDF-TrFE)/BaTiO_3_ scaffold alone, peaking in defects treated with a combination of the scaffold, MSCs and PBM. The BS and BMD significantly increased, while Tb.Sp decreased when irradiation was applied in defects treated with the scaffold and MSCs. This showcased the synergistic effect of these three elements on bone repair, as the morphometric analysis considered only the bone tissue that formed on top of the scaffold. The positive effect of PBM as an adjunctive therapy to increase bone formation in calvarial defects treated with MSCs combined with P(VDF-TrFE)/BaTiO_3_ scaffold could be related to the PBM capacity of regulating the osteogenic and immunomodulatory potential of MSCs [29,56,57]. Although no histological differences were observed in the new bone tissue regardless of the treatments, the presence of multinucleated giant cells in contact with the scaffold suggests a foreign body reaction and calls for further investigations on the capacity of these cells to degrade P(VDF-TrFE)/BaTiO_3_ [58].

We demonstrated the viability of synthesizing a low-cost P(VDF-TrFE)/BaTiO_3_ scaffold using an electrospinning technique. We also showed that MSCs and PBM therapy acted synergistically when combined with the P(VDF-TrFE)/BaTiO_3_ scaffold to promote bone repair in rat calvarial defects. Indeed, this combination induced more bone formation than the scaffold alone or in combination with either MSCs or PBM. These findings highlight the need to combine different approaches to achieve complete regeneration of challenging bone defects and find avenues for further investigations into innovative therapies in the field of bone tissue engineering.

## Data Availability

The datasets used and/or analyzed during the current study are available from the corresponding author on reasonable request.
